# Phytochemical Analysis, Antioxidant, Antibacterial, Cytotoxic, and Enzyme Inhibitory Activities of *Hedychium flavum* Rhizome

**DOI:** 10.3389/fphar.2020.572659

**Published:** 2020-09-17

**Authors:** Minyi Tian, Xianghuan Wu, Tingya Lu, Xiaoge Zhao, Feng Wei, Guodong Deng, Ying Zhou

**Affiliations:** ^1^ Key Laboratory of Plant Resource Conservation and Germplasm Innovation in Mountainous Region (Ministry of Education), Collaborative Innovation Center for Mountain Ecology & Agro-Bioengineering (CICMEAB), College of Life Sciences/Institute of Agro-bioengineering, Guizhou University, Guiyang, China; ^2^ Guizhou Engineering Center for Innovative Traditional Chinese Medicine and Ethnic Medicine, Guizhou University, Guiyang, China; ^3^ College of Pharmacy, Guizhou University of Traditional Chinese Medicine, Guiyang, China

**Keywords:** *Hedychium flavum* Roxb., phytochemical analysis, antioxidant, antibacterial, cytotoxic, enzyme inhibitory

## Abstract

*Hedychium flavum* Roxb., a medicinal, edible, and ornamental plant, is widely cultivated throughout China, India, and Southeast Asia. The rhizome from this plant has been used for food flavoring and in traditional Chinese medicine to treat diverse diseases, but the detailed constituents and bioactivities are still limited known. Therefore, phytochemical analysis by GC-MS and UHPLC-Q-Orbitrap-MS, and antioxidant, antibacterial, cytotoxic, and enzyme inhibitory activities tests have been conducted in the current study. Based on the GC-MS results, the essential oil (EO) of rhizome was mainly composed of coronarin E (20.3%), *β*-pinene (16.8%), *E*-nerolidol (11.8%), and linalool (8.5%). Among them, coronarin E was reported in *H. flavum* EO firstly. Furthermore, the spectrophotometric indicated rhizome had high total phenolic content (TPC, 50.08–57.42 mg GAEs/g extract) and total flavonoid content (TFC, 12.45–21.83 mg REs/g extract), no matter in water extract (WE) or in 70% ethanol extract (EE). UHPLC-Q-Orbitrap-MS was applied to further characterize composition, and 86 compounds were putatively identified from WE and EE, including 13 phenolic components. For the bioactivities, both WE and EE showed remarkable antioxidant activity by DPPH and ABTS tests, being superior to the positive control (butylated hydroxytoluene, BTH). EO revealed significant antibacterial activity against *Staphylococcus aureus*, *Bacillus subtilis*, *Pseudomonas aeruginosa*, and *Proteus vulgaris* with DIZ (10.34–24.43 mm), MIC (78.13–312.50 μg/mL), and MBC (156.25–625.00 μg/mL). Moreover, EO exhibited a considerable selectivity to human tumor cell K562 (IC_50_ = 27.16 μg/mL), and its toxicity was more than 3.5-fold different from that of non-cancerous MRC-5 cell (IC_50_ = 95.96 μg/mL) and L929 cell (IC_50_ = 129.91 μg/mL). A series of apoptosis analysis demonstrated that EO induced apoptosis against K562 cells in a dose-dependent manner. In enzyme inhibitory effect assays, WE and EE showed strong *α*-glucosidase inhibition activity, being superior to the positive control (acarbose). Besides, the EO, WE, and EE didn’t show a promising inhibition on tyrosinase (19.30–32.51 mg KAEs/g sample) and exhibited a weak inhibitory effect on cholinesterase. Based on the current results, *H. flavum* could be considered as a source of bioactive compounds and has high exploitation potential in the cosmetics, food, and pharmaceutical industries.

## Introduction

The *Hedychium* genus (Zingiberaceae), commonly known as ginger lily, includes approximately 87 species, which are mainly distributed in China, India, and Southeast Asia (The Plant List, 2010; Prakash et al., 2016). *Hedychium* species are extensively cultivated for their various uses in fragrance, paper, ornamental, cosmetics, medicine, and food industries (Sakhanokho and Rajasekaran, 2019). *Hedychium* plants are rich in essential oil and widely used in traditional medicine for the treatment of ﬂu, gastric diseases, diarrhea, bronchitis, asthma, nausea, snake bites, and leishmaniasis (Tushar et al., 2010; Hartati et al., 2014; Sakhanokho and Rajasekaran, 2019). The extracts and essential oils of *Hedychium* plants have been demonstrated to have multiple bioactivities, such as antioxidant, antimicrobial, cytotoxicity, anti-acetylcholinesterase, anti-inflammatory, antidiabetic, analgesic, hepatoprotective, and insecticidal activities (Hartati et al., 2014; Ray et al., 2018a; Tavares et al., 2020).

*Hedychium flavum* Roxb., a perennial plant with aromatic and tuberose characters, is widely cultivated for its aromatic essential oil (EO) and as a medicinal, edible, and ornamental plant in China, India, and Southeast Asia (Wu and Larsen, 2000; Thanh et al., 2014). The young tender shoots of *H. flavum* are used as vegetable and food flavorings (Devi et al., 2014). The EO from this plant flower has been used as spice and to treat gastric diseases (Pan and Zhang, 2013). The fresh rhizome of *H. flavum* is sliced or minced and used as food flavoring in Southwestern China. The rhizome has been used in Chinese folk medicine, commonly known as *yehansu*, to treat cough, cold, rheumatism, headache, abdominal pain, and bruise (CHMC-Chinese Herbal Medicine Company, 1994; Wang, 2010). Additionally, The EO from rhizome has been used in delicate, high-quality perfumes and traditional medicines (Prakash et al., 2016). According to previous studies, the main EO constituents of rhizome were *α*-pinene (4.3–12.1%), *β*-pinene (21.8–24.77%), 1,8-cineole (8.90–28.3%), and linalool (6.29–17.5%) (Sakhanokho et al., 2013; Thanh et al., 2014; Ray et al., 2018a). The major compounds of EO in leaf were *β*-pinene (22.5%), *α*-humulene (15.7%), and *β*-caryophyllene (10.4%), as well as the main EO components in stem were *α*-humulene (18.9%), *β*-caryophyllene (11.8%), and *β*-pinene (11.2%) (Thanh et al., 2014). Besides, the predominant EO compounds in aerial parts of *H. flavum* were *β*-pinene (49.6%), *β*-caryophyllene (26.9%), *α*-pinene (5.9%), sabinene (4.2%), and *α*-humulene (2.3%) (Mollenbeck et al., 1997). The EO from *H. flavum* rhizome has been demonstrated to possess antifungal and insecticidal activities (Rajasekaran et al., 2012; Sakhanokho et al., 2013).

*H. flavum* has been developed as an industrial crop with medicinal, ornamental, and spice values. However, in addition to few reports on the chemical components, antifungal and insecticidal activities of EO, there is no detailed study on the chemical composition and bioactivities of *H. flavum*, which could impede its exploitation in industry. Therefore, phytochemical analysis by GC-MS and UHPLC-Q-Orbitrap-MS, and antioxidant, antibacterial, cytotoxic, and enzyme inhibitory activities tests of the rhizome from *H. flavum* were conducted in this work. To our knowledge, the antioxidant, antibacterial, cytotoxic, and enzyme inhibitory activities of EO, as well as the chemical composition and bioactivities of extracts from *H. flavum* rhizome were reported for the first time.

## Materials and Methods

### Chemical and Reagents

Gallic acid, rutin, streptomycin, cisplatin, acarbose, kojic acid, galanthamine, resazurin, and Folin-Ciocalteu reagent were obtained from Solarbio Life Sciences (Beijing, China). Ascorbic acid, butylated hydroxytoluene (BHT), 1,1-diphenyl-2-picrylhydrazyl (DPPH), 2,2-azino-bis-3-ethylbenzthiazoline-6-sulphonic acid (ABTS), acridine orange (AO), ethidium bromide (EB), α-glucosidase, tyrosinase, acetylcholinesterase (AChE), butyrylcholinesterase (BChE), p-Nitrophenyl-α-D-glucopyranoside (p-NPG), L-tyrosine, acetylthiocholine iodide (ATCI), and butyrylthiocholine chloride (BTCl) were purchased from Sigma-Aldrich (Germany). Hoechst 33258 and Annexin V-FITC/PI Apoptosis Detection Kit were from Beyotime (Haimen, China). For GC-MS analysis, analytical standard n-alkanes (C_8_–C_22_) were from Merck (Darmstadt, Germany). For UPLC-MS analysis, LC/MS-grade acetonitrile, formic acid, and ultrapure water were supplied from Merck (Darmstadt, Germany). All other chemicals and solvents were of analytical grade and purchased from Aladdin (Shanghai, China).

### Plant Material

*H. flavum* rhizome was collected in April 2019 from Guangxi Province, China. The specie was identified by Prof. Guoxiong Hu of Guizhou University. The voucher specimen was deposited at Guizhou Engineering Center for Innovative Traditional Chinese Medicine and Ethnic Medicine, Guizhou University (Voucher No: HF-20190408).

### Preparation of EO, WE, and EE

To produce EO, the fresh, finely chopped rhizomes (1.5 kg) were placed into a Clevenger-type apparatus and hydrodistilled (4 h). EO was dried over anhydrous Na_2_SO_4_, filtered and stored at 4°C.

WE and EE were prepared by reflux extraction method. The fresh, finely chopped rhizomes (0.5 kg) were placed into a round bottom flask (2 L), mixed with distilled water or 70% ethanol, boiled under reflux for 2 h, and then filtered. The reflux extraction was repeated twice. The combined filtrate was concentrated with a rotary evaporator and dried in a freeze-drier. Then, WE and EE were weighed and stored at 4°C.

### GC- FID/MS for Composition Analysis of EO

The EO was analyzed by an Agilent 6890 gas chromatograph equipped with a flame ionization detector (FID). Capillary column: HP-5MS (60 m × 0.25 mm, 0.25 μm film thickness). The flow rate of carrier gas helium was set at 1 mL/min and the split ratio was 1:20. The following GC oven temperature was used with the injection volume of 1 μL: held at 70°C (2 min), 2°C per min to 180°C (55 min), 10°C per min to 310°C (13 min), and kept at 310°C (12 min). The GC-MS analysis was performed using an Agilent 6890 gas chromatograph equipped with an Agilent 5975C mass selective detector (Agilent Technologies Inc., CA, USA). GC column and parameters were the same as in GC-FID. The mass spectra operated in the mass range (*m/z* 29 to 500) and EI mode (70 eV). The interface temperature and ion source temperature were 280°C and 230°C, respectively. The relative percentage of chemical constituents was determined by the peak area. The retention index (RI) is determined by referring to a series of n-alkanes (C_8_–C_22_). The identification of chemical constituents was based on comparison of their retention index and mass spectrum with those in Wiley 275 and NIST 2017 databases.

### Total Phenolic and Flavonoid Contents

#### Total Phenolic Content (TPC)

The TPC was evaluated according to Folin-Ciocalteu method (Slinkard and Singleton, 1977) with marginal modification. The sample solution (0.5 mL) was mixed with Folin-Ciocalteu reagent (2.5 mL) and incubated for 4 min. Subsequently, Na_2_CO_3_ solution (4 mL, 7.5%) was added and incubated at room temperature for 60 min. The sample absorbance was read using a UV-5200 spectrophotometer (Shanghai Metash Instruments Co., Ltd, China) at 760 nm. TPC was expressed as equivalents of gallic acid (mg GAEs/g extract).

#### Total Flavonoid Content (TFC)

The TFC was assayed by NaNO_2_-Al(NO_3_)_3_-NaOH colorimetry (China Pharmacopoeia Commission, 2015) with minor modification. The sample solution (5 mL) was mixed with 5% NaNO_2_ (0.4 mL) and incubated for 6 min. Subsequently, Al(NO_3_)_3_ solution (0.4 mL, 10%) was added and incubated for 6 min. Then, NaOH solution (4 mL, 4%) was added up to a total volume of 10 mL with distilled water. The absorbance was read at 510 nm after 15 min incubation at room temperature. TFC was expressed as rutin equivalents (mg REs/g extract).

### UHPLC-Q-Orbitrap-MS Analysis of WE and EE Components

The chemical components of WE and EE were determined using ultra-high-performance liquid chromatography coupled to quadrupole-Orbitrap high-resolution mass spectrometry (UHPLC-Q-Orbitrap-MS). Chromatographic separation was carried out using a Dionex Ultimate 3000 UPLC System (Thermo Fisher Scientific, USA), with a Hypersil GOLD aQ (2.1 mm × 100 mm, i.d. 1.9 μm, Thermo Fisher Scientific) column. Mobile phases A and B were formic acid water (0.1%, v/v) and formic acid acetonitrile (0.1%, v/v), respectively. The flow rate was 0.3 mL/min. The gradient was as follows: 0–2 min, 5% B; 2–42 min, 5–95% B; 42–47 min, 95% B; 47.1 min, 5% B; 47.1–50 min, 5% B. The injection volume was 5 μL and the column temperature was held at 40°C.

The Thermo Scientific Q Exactive Focus hybrid quadrupole-Orbitrap mass spectrometry (Q-Orbitrap-MS) equipped with heated electrospray ionization (HESI-II) source was used for MS data collection. HESI-II source was operated in negative and positive ionization modes with spray voltages of 2.5 and 3.0 kV, respectively. HESI-II source parameters were set as follows: capillary temperature 320°C, vaporizer temperature 350°C, sweep gas pressure 0 arb, auxiliary gas pressure 10 arb, sheath gas pressure 35 arb, RF lens amplitude (S-lens) 60. The mass spectrometer analysis was executed in the mode of full mass/dd-MS^2^, scanned from *m/z* 100 to 1,500 with a resolution of 70,000 in full scan MS^1^, followed by data-dependent MS^2^ acquisition at 17,500 resolution with stepped normalized collision energy (NCE) (20, 40, and 60 eV). Maximum injection time (IT) for MS^1^ and MS^2^ was separately set at 100 and 50 ms and the target values of automatic gain control (AGC) were 1e^6^ and 2e^5^, respectively. Loop count was 3, dynamic exclusion time was 5 s, and quadrupole isolation window (isolation width) was 1.5 *m/z*. The mass spectrum data of the chemical composition was processed with CD3.0 (Thermo Fisher Scientific) and identified by comparing mzVault, mzCloud, and ChemSpider databases. The allowable calculation error was set as 5 ppm.

### Antioxidant Activity

#### DPPH Assay

The 1,1-diphenyl-2-picrylhydrazyl (DPPH) radical scavenging activity was evaluated according to the method with slight modification (Blois, 1958). Two milliliter of sample solution and DPPH solution (2 mL, 0.1 mM) were mixed and incubated for 30 min at room temperature in the dark. The absorbance was recorded at 517 nm. Butylated hydroxytoluene (BHT) and ascorbic acid were used as positive controls. The results were expressed using IC_50_ values and as equivalents of ascorbic acid (mg AEs/g sample).

#### ABTS Assay

The 2,2-azino-bis-3-ethylbenzthiazoline-6-sulphonic acid (ABTS) radical scavenging capacity was assayed according to the method with marginal modification (Re et al., 1999). The ABTS•^+^ solution was generated by reacting 2.45 mM K_2_S_2_O_8_ solution with and 7 mM ABTS solution incubated in the dark at room temperature for 12 h. Before experiments, to obtain an absorbance of 0.70 ± 0.02 at 734 nm, the ABTS•^+^ solution was diluted with methanol. The diluted ABTS•^+^ solution (4 mL) and sample solution (0.4 mL) were mixed and the absorbance was read at 734 nm after 10 min incubation in the dark at room temperature. Ascorbic acid and BHT were used as positive controls. The results were expressed using IC_50_ values and as equivalents of ascorbic acid (mg AEs/g sample).

### Antibacterial Activity

#### Bacterial Strains

The antibacterial capacity of EO, WE, and EE was evaluated performed against the following bacterial strains: *Proteus vulgaris* (CMCC (B) 49027), *Escherichia coli* (ATCC 25922), *Bacillus subtilis* (CMCC (B) 63501), *Pseudomonas aeruginosa* (CMCC (B) 10104), *Enterococcus faecalis* (ATCC 29212), and *Staphylococcus aureus* (ATCC 6538P).

#### Agar Well Diffusion Assay

The inhibition zone diameter was assayed according to the agar well diffusion method with slight modification (Zhang et al., 2017). Briefly, The WE and EE were dissolved with distilled water (100 mg/mL). Streptomycin distilled water solution (1 mg/mL) was used as the positive control. The bacterial suspension (100 μL, 10^6^ CFU/mL) was evenly spread on the Mueller-Hinton agar medium. Filter paper discs (diameter 6 mm) containing pure essential oil, WE, EE, and streptomycin solution (20 μL) were added and the diameter of inhibition zone (DIZ) was recorded after 24 h incubation at 37°C.

#### Determination of MIC and MBC

The minimal inhibitory concentration (MIC) and minimal bactericidal concentration (MBC) values were determined by the broth microdilution method with marginal modification (Eloff, 1998). Briefly, the two-fold serially diluted sample solution (100 μL) and the bacterial suspensions (100 μL) were added to each well at a final density of 5 × 10^5^ CFU/mL. The 96-well plates were incubated for 24 h at 37°C. Subsequently, the resazurin aqueous solution (10 μL, 0.01%) was added to each well and incubated for 2 h at 37°C in the dark. The MIC was determined as the minimum sample concentration without color change. For the determination of MBC value, the sample (10 μL) from the wells without color change was subcultured in Mueller Hinton agar plate for 24 h at 37°C. The MBC was defined as the minimum sample concentration showing no bacterial growth (Mzid et al., 2017).

### Cytotoxic Activity

The cytotoxic effect was assayed according to our previously published MTT method (Tian et al., 2020) against murine fibroblast cell line (L929) and five human cell lines: fetal lung fibroblasts (MRC-5), lung adenocarcinoma (A549), and non-small cell lung cancer (NCI-H1299), prostatic carcinoma (PC-3), and leukemic (K562) cell lines. EO solution (0.1% DMSO), WE, and EE distilled water solution were two-fold serially diluted with medium. The cell suspensions (80 μL) were added to each well at a density of 5 × 10^3^ cells per well and incubated for 24 h. Subsequently, 20 μL of the diluted sample solution was added and cultured for 72 h. After that, the MTT solution (10 μL, 5 mg/mL in PBS) was added and incubated for 4 h. Then, 150 μL of DMSO was added to each well to dissolve formazan crystal after removing the medium. The absorbance was recorded using an iMark microplate reader (Bio-Rad Laboratories, Inc., Hercules, CA, USA) at 490 nm. The results were expressed using IC_50_ values.

### Apoptosis Analysis

#### Morphology Assay

The K562 cell suspension was added to a 6-well plate (1×10^6^ cells per well) and incubated for 24 h. Subsequently, the cells were treated with varying concentrations of EO (0, 30, and 60 μg/mL) for 48 h. Morphological changes of the treated cells were recorded using an inverted microscope (Leica DMi8, Leica Microsystems, Germany).

For Acridine orange/ethidium bromide (AO/EB) staining assay, the cells were treated with EO as described above. The treated cell suspensions (1 mL) were collected, centrifuged at 500 g for 10 min, and resuspended in 50 μL PBS, then stained with AO (10 µg/mL) and EB (10 µg/mL) for 10 min. After that, the stained cell suspension (10 μL) was placed onto a glass slide and photographed using an inverted fluorescence microscope (Leica DMi8, Leica Microsystems, Germany).

For Hoechst 33258 staining assay, the cells were treated with EO as described above. The treated cell suspensions (1 mL) were collected, centrifuged, and resuspended in 1 mL PBS. After removing the PBS, the cells were fixed in 4% paraformaldehyde (10 μL) for 10 min. After two PBS washes, the cells were stained with 10 μL of Hoechst 33258 (Beyotime, Haimen, China) for 10 min. After that, the stained cell suspension (10 μL) was placed onto a glass slide and analyzed by an inverted fluorescence microscope.

#### Annexin V-FITC/PI Assay

Annexin V-FITC and propidium iodide (PI) staining was performed using flow cytometry to quantify EO-induced apoptosis in K562 cell. The K562 cells were seeded in 6-well plates at 2×10^5^/well, and treated with different concentrations of EO (0, 30, and 60 μg/mL) for 48 h. Subsequently, the cells were collected and stained with Annexin V-FITC/PI as described by the manufacturer using Annexin V-FITC/PI Apoptosis Detection Kit (Beyotime, Haimen, China). The labeled cells were analyzed using a BD FACSCalibur flow cytometer (BD Bioscience, Franklin Lakes, NJ, USA).

### Enzyme Inhibitory Activities

#### α-Glucosidase Inhibitory Activity

The α-glucosidase inhibitory activity was evaluated according to the method with slight modification (Silva et al., 2019). Sample solution (30 μL), phosphate buffer (60 μL, pH 6.8), and α-glucosidase solution (10 μL, 0.8 U/mL) were mixed and added to each well. After 15 min incubation at 37°C, p-Nitrophenyl-α-D-glucopyranoside (p-NPG) solution (10 μL, 1 mM) was added and incubated at 37°C for 15 min. Subsequently, the reaction was stopped with the addition of Na_2_CO_3_ solution (80 μL, 0.2 M), and the absorbance was recorded at 405 nm. Acarbose was used as positive control. The α-glucosidase inhibitory effect was expressed using IC_50_ values and as equivalents of acarbose (mmoL ACEs/g sample).

#### Tyrosinase Inhibitory Activity

The inhibition of tyrosinase was assayed according to the method with L-tyrosine as substrate (Zardi-Bergaoui et al., 2018). Sample solution (70 μL) and tyrosinase solution (10 μL, 100 U/mL) were mixed in a 96-well plate and incubated at 37°C for 5 min. Then, L-tyrosine solution (80 μL, 5.5mM) was added and incubated at 37°C for 30 min. Kojic acid was used as positive reference, and the absorbance was recorded at 492 nm. The tyrosinase inhibitory activity was expressed using IC_50_ values and as equivalents of kojic acid (mg KAEs/g sample).

#### Cholinesterase Inhibition Activity

The inhibitory effects of cholinesterase including acetylcholinesterase (AChE) and butyrylcholinesterase (BChE) were evaluated according to Ellman’s method with slight modification (Ellman et al., 1961). Sample solution (50 μL) was mixed AChE or BuChE solution (pH 8.0, 10 μL, 0.5 U/mL) in a 96-well plate and incubated at 4°C for 15 min. Subsequently, acetylthiocholine iodide (ATCI) or butyrylthiocholine chloride (BTCl) solution (20 μL, 2mM) and 5,5′-dithiobis-(2-nitrobenzoic acid) (DTNB) solution (20 μL, pH 8.0, 2 mM) were added and incubated at 37°C for 30 min. After that, the reaction was stopped by adding 20 μL of 0.018 mM physostigmine, and finally the absorbance was read at 405 nm. Galanthamine was used as a positive reference. The cholinesterase inhibitory effects were expressed using IC_50_ values and as equivalents of galanthamine (mg GALAEs/g sample).

### Statistical Analysis

All data were repeated at least three times and expressed as mean ± standard deviation (SD). SPSS software (version 19.0) was used for statistical analysis. The significant difference between the two groups was compared using the two-tailed unpaired t-test (*P* < 0.05).

## Results and Discussion

### Chemical Composition of EO

**Figure 1:**
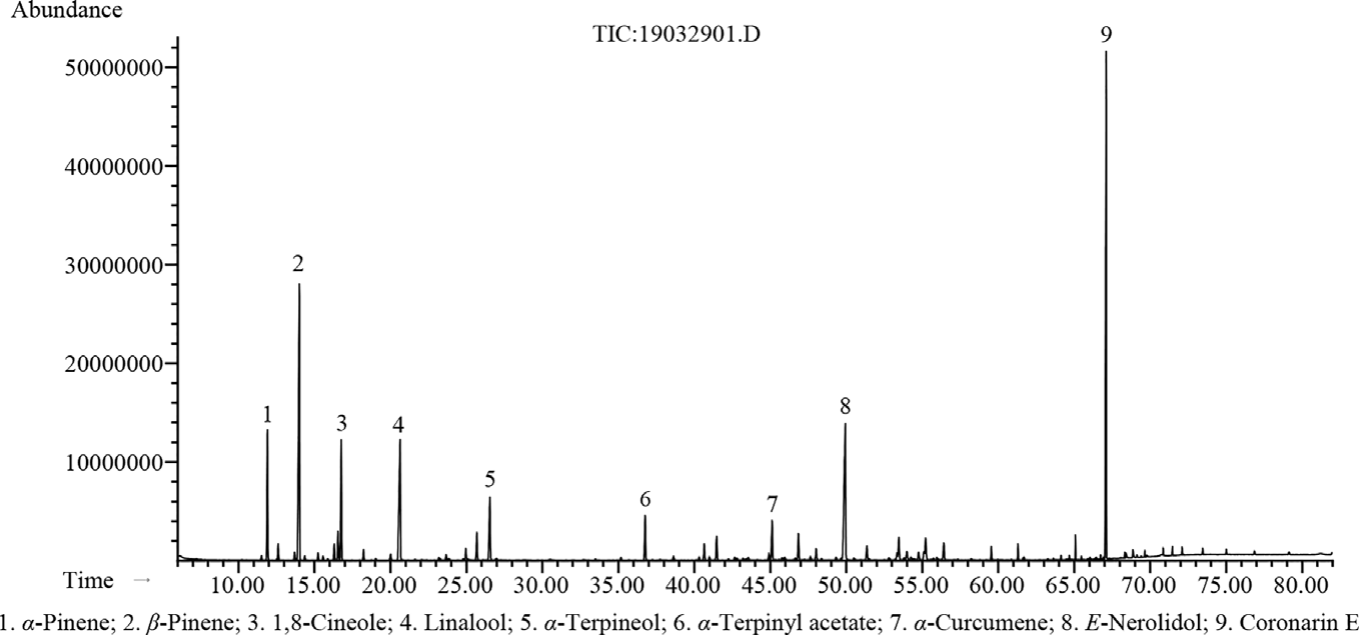
GC-MS chromatogram of *H. flavum* EO.

The hydrodistillation of *H. flavum* fresh rhizomes yielded EO at 0.56% (w/w) on fresh weight basis. Seventy-seven chemical constituents of EO were identified by GC-MS/FID, representing 96.5% of the composition (**Table 1**). The major constituents of *H. flavum* EO were coronarin E (20.3%), *β*-pinene (16.8%), *E*-nerolidol (11.8%), linalool (8.5%), 1,8-cineole (5.5%), *α*-pinene (5.4%), *α*-terpineol (3.8%), *α*-terpinyl acetate (2.4%), and *α*-curcumene (2.0%), which were presented in **Figure 1**. Previous studies have showed that the main compounds of *H. flavum* rhizome EO were *α*-pinene (4.3–12.1%), *β*-pinene (21.8–24.77%), 1,8-cineole (8.90–28.3%), and linalool (6.29–17.5%) (Sakhanokho et al., 2013; Thanh et al., 2014; Ray et al., 2018a). Furthermore, the main EO components in leaf were *β*-pinene (22.5%), *α*-humulene (15.7%) and *β*-caryophyllene (10.4%), as well as the major EO compounds of stem were *α*-humulene (18.9%), *β*-caryophyllene (11.8%), and *β*-pinene (11.2%) (Thanh et al., 2014). The main EO components in aerial parts of *H. flavum* were *β*-pinene (49.6%), *β*-caryophyllene (26.9%), *α*-pinene (5.9%), sabinene (4.2%), and *α*-humulene (2.3%) (Mollenbeck et al., 1997). However, in our study, the coronarin E is the most predominant constituent, which has not been detected in previous *H. flavum* EO studies. The coronarin E has previously been reported in other *Hedychium* species EO, such as *H. coronarium* (Rodrigues et al., 2013; Ray et al., 2018b; Ray et al., 2019), *H. acumnatum* (Weyerstahl et al., 1995) and *H. gardnerianum* (Weyerstahl et al., 1998). To our knowledge, the coronarin E, as the most predominant constituent, was identified for the first time in *H. flavum* EO.

**Table 1:** Chemical components of the essential oil from *H. flavum* rhizome.

Compounds	RI^a^	RI^b^	RT (min)	% Area	Identification^c^
Octane	800	800	7.23	tr^d^	MS, RI
Tricyclene	926	925	11.43	tr^d^	MS, RI
*α*-Thujene	928	929	11.52	0.2	MS, RI
*α*-Pinene	937	937	11.91	5.4	MS, RI
*α*-Fenchene	950	950	12.51	tr^d^	MS, RI
Camphene	953	952	12.62	0.7	MS, RI
Sabinene	976	974	13.69	0.4	MS, RI
*β*-Pinene	983	979	14.01	16.8	MS, RI
6-Methyl-5-hepten-2-one	986	986	14.13	tr^d^	MS, RI
*β*-Myrcene	991	991	14.36	0.2	MS, RI
*α*-Phellandrene	1,008	1,005	15.24	0.3	MS, RI
*δ*-3-Carene	1,014	1,011	15.57	0.2	MS, RI
*α*-Terpinene	1,020	1,017	15.88	0.1	MS, RI
*p*-Cymene	1,027	1,023	16.30	0.7	MS, RI
*α*-Limonene	1,031	1,030	16.55	1.4	MS, RI
1,8-Cineole	1,035	1,032	16.76	5.5	MS, RI
*trans*-Ocimene	1,047	1,047	17.47	tr^d^	MS, RI
*γ*-Terpinene	1,060	1,060	18.23	0.5	MS, RI
*trans*-Sabinene hydrate	1,069	1,070	18.74	tr^d^	MS, RI
*cis*-Linalool oxide	1,075	1,074	19.03	0.1	MS, RI
*α*-Terpinolene	1,092	1,088	20.02	0.4	MS, RI
Linalool	1,102	1,099	20.64	8.5	MS, RI
Nopinone	1,142	1,137	23.19	0.1	MS, RI
*trans*-Pinocarveol	1,144	1,139	23.27	0.1	MS, RI
Camphor	1,150	1,145	23.67	0.3	MS, RI
Camphene hydrate	1,153	1,148	23.88	tr^d^	MS, RI
Pinocarvone	1,168	1,164	24.80	0.1	MS, RI
Borneol	1,170	1,167	24.96	0.7	MS, RI
Terpinen-4-ol	1,182	1,182	25.69	1.4	MS, RI
*p*-Cymen-8-ol	1,188	1,183	26.10	tr^d^	MS, RI
Cryptone	1,191	1,184	26.30	tr^d^	MS, RI
*α*-Terpineol	1,195	1,189	26.55	3.8	MS, RI
Myrtenol	1,201	1,195	26.94	tr^d^	MS, RI
Myrtenal	1,202	1,193	26.99	0.1	MS, RI
*cis*-Geraniol	1,230	1,228	28.79	0.1	MS, RI
*trans*-Geraniol	1,255	1,255	30.44	tr^d^	MS, RI
l-Bornyl acetate	1,290	1,284	32.72	tr^d^	MS, RI
Isobornyl acetate	1,292	1,286	32.84	tr^d^	MS, RI
Carvacrol	1,302	1,299	33.49	0.1	MS, RI
*α*-Terpinyl acetate	1,353	1,350	36.76	2.4	MS, RI
*α*-Copaene	1,382	1,376	38.63	0.2	MS, RI
Sesquithujene	1,409	1,402	40.32	0.2	MS, RI
Isocaryophyllene	1,415	1,406	40.65	0.9	MS, RI
*cis*-*α*-Bergamotene	1,420	1,415	40.99	0.2	MS, RI
*β*-Caryophyllene	1,428	1,419	41.47	1.3	MS, RI
*γ*-Elemene	1,439	1,433	42.15	tr^d^	MS, RI
*trans*-*α*-Bergamotene	1,441	1,435	42.25	0.1	MS, RI
Guaia-6,9-diene	1,450	1,443	42.81	0.1	MS, RI
*trans*-*β*-Farnesene	1,459	1,453	43.37	0.1	MS, RI
*α*-Humulene	1,462	1,454	43.55	0.1	MS, RI
*γ*-Curcumene	1,484	1,480	44.91	0.3	MS, RI
*α*-Curcumene	1,487	1,483	45.12	2.0	MS, RI
*β*-Selinene	1,491	1,489	45.35	tr^d^	MS, RI
*δ*-selinene	1,495	1,492	45.56	tr^d^	MS, RI
*α*-selinene	1,498	1,498	45.76	0.1	MS, RI
Isohomogenol	1,499	1,492	45.84	0.1	MS, RI
*trans*-Dihydroagarofuran	1,510	1,496	46.49	tr^d^	MS, RI
*β*-Bisabolene	1,513	1,509	46.65	0.1	MS, RI
*β*-Curcumene	1,516	1,514	46.85	1.4	MS, RI
7-epi-*α*-Selinene	1,527	1,517	47.46	tr^d^	MS, RI
*δ*-Cadinene	1,530	1,524	47.64	0.2	MS, RI
*D*-Nerolidol	1,536	1,544	48.01	0.6	MS, RI
Italicene ether	1,542	1,544	48.37	0.1	MS, RI
*cis*-Sesquisabinene hydrate	1,559	1,542	49.33	0.2	MS, RI
*E*-Nerolidol	1,569	1,564	49.94	11.8	MS, RI
Caryophyllene oxide	1,593	1,581	51.35	0.8	MS, RI
*γ*-Eudesmol	1,631	1,631	53.46	1.3	MS, RI
Isospathulenol	1,637	1,638	53.81	0.1	MS, RI
Agarospirol	1,645	1,645	54.25	0.2	MS, RI
*β*-Eudesmol	1,660	1,649	55.11	0.4	MS, RI
Valerianol	1,662	1,661	55.23	1.4	MS, RI
*β*-Bisabolol	1,676	1,671	55.96	0.1	MS, RI
Ambrial	1,817	1,809	61.30	0.5	MS, RI
2-Heptadecanone	1,903	1,902	63.26	tr^d^	MS, RI
(*E*)-15,16-Dinorlabda-8(17),11-dien-13-one	2,010	1,994	65.08	0.6	MS, RI
Geranyl linallol	2,038	2,034	65.47	0.1	MS, RI
Coronarin E	2,166	2,136	67.10	20.3	MS, RI
Total identified				96.5	

a Retention index (RI) was determined by referring to a series of n-alkanes (C8–C22) on the HP-5MS column.

b Retention index (RI) from Wiley 275 and NIST 2017 databases.

c Identification: based on the comparison of their RI and MS with those in Wiley 275 and NIST 2017 databases.

d tr: trace (trace < 0.1%).

### Total Phenolic and Flavonoid Contents

**Figure 2:**
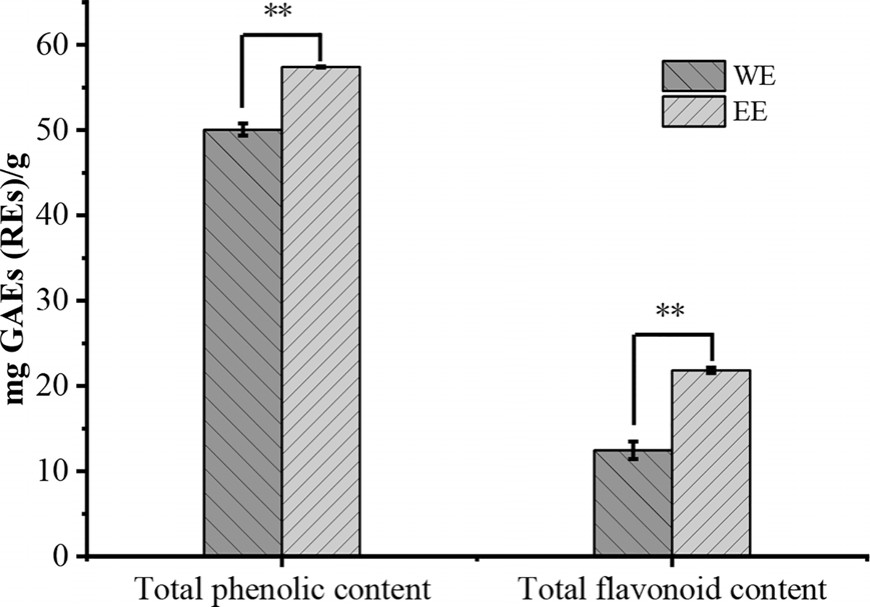
Total phenolic and flavonoid contents of WE and EE, ^**^ *p* < 0.01.

The WE presented a higher yield at 2.04% (w/w) on fresh weight basis than the EE (1.37%). The TPC and TFC of *H. flavum* rhizome WE and EE were shown in **Figure 2**. The TPC of WE and EE were 50.08 ± 0.71 and 57.42 ± 0.07 gallic acid equivalents (mg GAEs/g) of dry weight of extract, respectively. The TFC in WE and EE were 12.45 ± 1.01 and 21.83 ± 0.33 rutin equivalents (mg REs/g) of dry weight of extract, respectively. Statistical analysis indicated that TPC and TFC of EE were significantly higher (*p*< 0.01) than that of WE. Plant polyphenols and flavonoids have been widely used in various medicines and food products due to their wide range of pharmacological activities, such as antioxidants, antimicrobial, anticancer, anti-inflammatory, hepatoprotective, antiviral, antiatherosclerotic, antispasmodic, and enzyme inhibitory activities (Fraga, 2010; Agrawal, 2011; Murti and Sharma, 2017). The *H. flavum* rhizome WE and EE exhibited high TPC and TFC values, which can be used as a rich source of phenolics and flavonoids.

### Identification of WE and EE Compounds by UHPLC-Q-Orbitrap-MS

**Figure 3:**
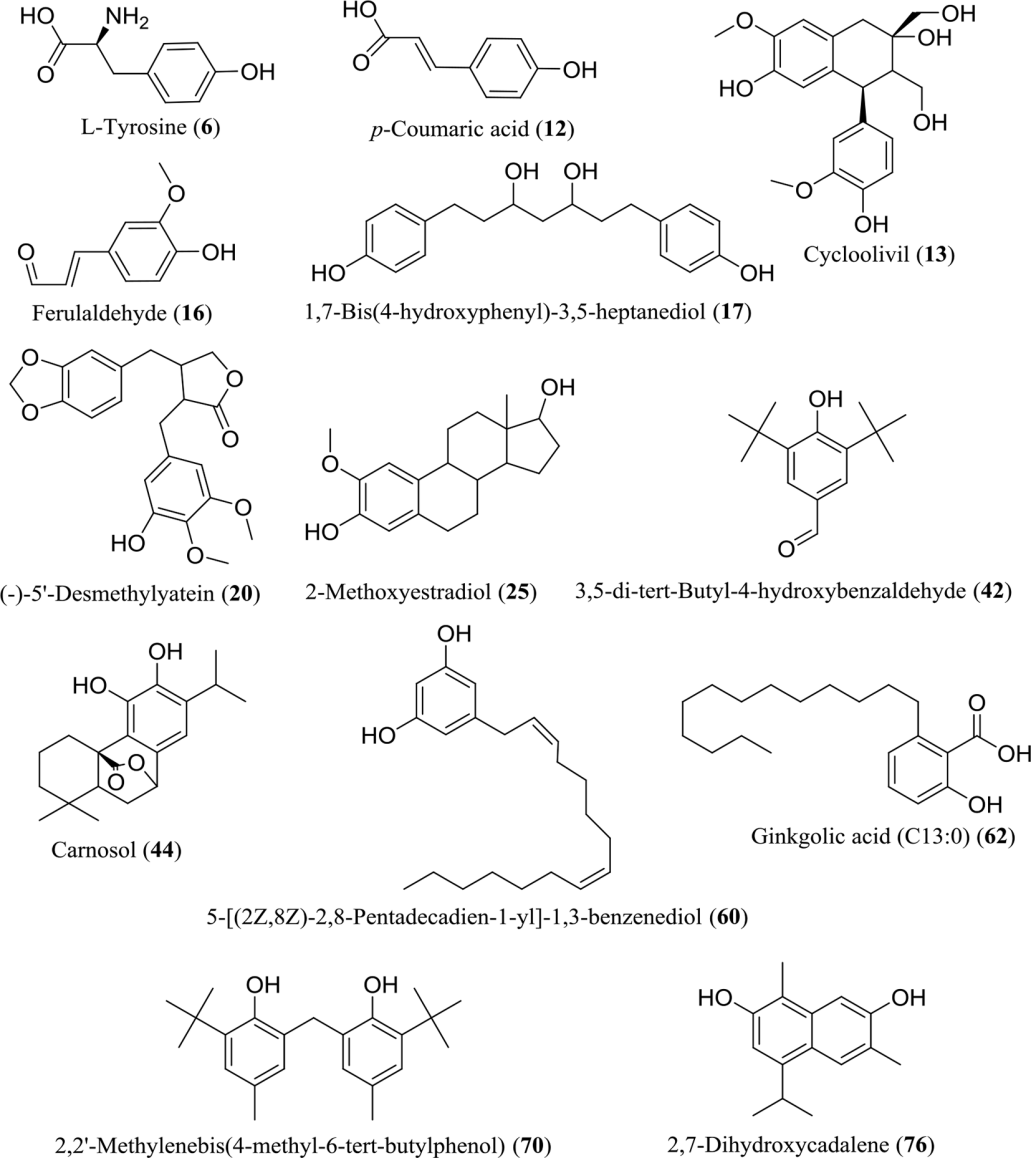
Chemical structures of 13 identified phenolic compounds in WE and EE.

The WE and EE compounds were investigated by UHPLC-Q-Orbitrap-MS using negative and positive ion mode scanning. According to the primary and secondary high-resolution mass spectrometry (MS^1^/MS^2^) and comparative analysis using mzCloud, ChemSpider, and mzCloud databases, a total of 86 compounds were putatively identified (**Supplementary Material, Table S1**). The WE and EE showed differentiation in chemical composition (**Supplementary Material, Figure S1**). Seventy-one compounds in WE and forty-three components in EE were identified, respectively. Considering the high TPC value of WE and EE, 13 identified phenolic compounds were listed in **Figure 3**, including L-tyrosine (6), *p*-coumaric acid (12), cycloolivil (13), ferulaldehyde (16), 1,7-bis(4-hydroxyphenyl)-3,5-heptanediol (17), (-)-5’-desmethylyatein (20), 2-methoxyestradiol (25), 3,5-di-tert-butyl-4-hydroxybenzaldehyde (42), carnosol (44), 5-[(2Z,8Z)-2,8-pentadecadien-1-yl]-1,3-benzenediol (**60**), ginkgolic acid (C13:0) (62), 2,2’-methylenebis(4-methyl-6-tert-butylphenol) (70), and 2,7-dihydroxycadalene (76). All 86 compounds were identified from *H. flavum* for the first time.

### Antioxidant Activity

The antioxidant activity of EO, WE, and EE was evaluated using DPPH and ABTS methods, and BHT and ascorbic acid were used as positive controls (**Table 2**). The EO showed low antioxidant activity in both methods. The WE (IC_50_: 15.36 ± 0.15 μg/mL) and EE (IC_50_: 16.24 ± 0.61 μg/mL) exhibited significantly stronger DPPH free radical scavenging capacity than BHT (IC_50_: 109.40 ± 9.68 μg/mL), but lower than ascorbic acid (IC_50_: 9.39 ± 0.79 μg/mL) (*p* < 0.05). Base on ABTS analysis, the WE (IC_50_: 16.83 ± 0.45 μg/mL) and EE (IC_50_: 12.37 ± 0.13 μg/mL) showed significantly stronger antioxidant ability than both BHT (IC_50_: 18.86 ± 0.51 μg/mL), and ascorbic acid (IC_50_: 29.98 ± 1.77μg/mL) (*p* < 0.05). The free radical scavenging capacity of WE and EE was higher in ABTS assay (1783.16 ± 137.57 and 2424.29 ± 158.31, respectively) than in DPPH assay (611.13 ± 48.36 and 578.62 ± 54.10, respectively), expressed as ascorbic acid equivalents per gram of extract. Previous studies have shown that the DPPH and ABTS free radical scavenging capabilities of plant extracts were positively correlated with their TPC and TFC (Shin et al., 2015; Baloglu et al., 2019). Thus, the antioxidant capacity of WE and EE may be due to the high levels of TPC and TFC. The free radical scavenging agents can be used as a preventive medicine for age-related diseases and cancer (Khansari et al., 2009). The BHT, a synthetic antioxidant, is used as preservative in foods. However, as a food additive, the BHT is rejected by consumers due to its adverse reactions (Imaida et al., 1983; Simon, 2003). Interestingly, the WE and EE showed higher antioxidant activity compared with the BHT. These results indicate that WE and EE can be used as potential substitutes for synthetic antioxidants in the pharmaceutical and food industries. The authors limited the results of the DPPH analysis.

**Table 2:** Antioxidant activity of the EO, WE and EE from *H. flavum* rhizome.

Treatment	DPPH		ABTS		Yield (W/W)
	IC_50_ (μg/mL)^1^	mg AEs/g sample^2^	IC_50_ (μg/mL)^1^	mg AEs/g sample^2^	
EO	20653.02 ± 778.45^a^	0.47 ± 0.03^a^	1351.17 ± 16.89^a^	22.20 ± 1.48^a^	0.56%
WE	15.36 ± 0.15^b^	611.13 ± 48.36^b^	16.83 ± 0.45^b^	1783.16 ± 137.57^b^	2.04%
EE	16.24 ± 0.61^b^	578.62 ± 54.10^b^	12.37 ± 0.13^c^	2424.29 ± 158.31^c^	1.37%
Ascorbic acid^3^	9.39 ± 0.79^c^		29.98 ± 1.77^d^		
BHT^3^	109.40 ± 9.68^d^		18.86 ± 0.51^e^		

^a-e^Different letters in the same column represent significant difference (p < 0.05).

^1^IC_50_: The concentration of sample that affords a 50% free radical scavenging capacity in the assay.

^2^AEs: Ascorbic acid equivalents per gram of sample.

^3^ Ascorbic acid and BHT as positive control.

### Antibacterial Activity

The antibacterial properties of EO, WE, and EE were qualitatively determined by the diameter of inhibition zones (DIZ) and quantitatively evaluated by MIC and MBC values using streptomycin as positive control (**Table 3**). The antibacterial property of EO was higher than that of WE and EE. Both WE and EE displayed very low or no inhibition against the tested bacterial strains. The EO revealed significant antibacterial activity against *Pseudomonas aeruginosa*, *Bacillus subtilis*, *Staphylococcus aureus*, and *Proteus vulgaris* with DIZ values ranged from 10.34 to 24.43 mm, MIC values ranged from 78.13 to 312.50 μg/mL, and MBC values ranged from 156.25 to 625.00 μg/mL. The major constituents of *H. flavum* EO, such as coronarin E, *β*-pinene, *E*-nerolidol, linalool, *α*-terpineol, *α*-pinene, and 1,8-cineole, have been demonstrated to have antibacterial activity (Sokovic et al., 2008; Park et al., 2012; Hartati et al., 2015; Silva et al., 2015; Kiryu et al., 2018). Hence, *H. flavum* EO possessed significant antibacterial ability, probably due to these major constituents, and it can provide natural antibacterial agents for the pharmaceutical industry.

**Table 3:** Antibacterial activity of *H. flavum* rhizome EO, WE and EE.

Bacterial strains^a^	EO			WE	EE	Streptomycin		
	DIZ^b^	MIC^c^	MBC^c^	DIZ^b^	DIZ^b^	DIZ^b^	MIC^c^	MBC^c^
Gram positive								
*E. faecalis*	9.02 ± 1.18	625.00	2500.00	na	7.65 ± 0.45	8.44 ± 0.96	0.39	0.78
*S. aureus*	11.37 ± 1.49	312.50	625.00	7.63 ± 1.03	7.55 ± 0.92	17.83 ± 0.78	0.78	1.56
*B. subtilis*	18.68 ± 0.82	312.50	312.50	na	7.04 ± 0.93	18.65 ± 2.10	0.20	0.39
Gram negative								
*P. aeruginosa*	10.34 ± 2.50	312.50	625.00	9.27±1.00	na	12.52 ± 3.40	0.78	1.56
*E. coli*	8.73 ± 1.31	2500.00	2500.00	na	8.17 ± 0.87	16.40 ± 0.71	0.78	6.25
*P. vulgaris*	24.43 ± 5.04	78.13	156.25	6.92 ±0.53	7.45 ± 0.78	16.72 ± 0.77	0.39	0.39

The MIC and MBC of WE and EE were not listed in the table cause for being not detected at 2500 g/mL.

a Bacterial strains: Enterococcus faecalis (ATCC 29212), Staphylococcus aureus (ATCC 6538P), Bacillus subtilis (CMCC (B) 63501), Pseudomonas aeruginosa (CMCC (B) 10104), Escherichia coli (ATCC 25922), and Proteus vulgaris (CMCC (B) 49027).

b DIZ: The diameter of the inhibition zones (mm) including the diameter of the disk (6 mm). Sample solution: Pure EO (tested volume: 20 μL); WE and EE distilled water solution (tested volume: 20 μL, 100 mg/mL); Positive control: Streptomycin (tested volume: 20 μL, 1 mg/mL). Na, not active.

c MIC: Minimal inhibitory concentration (μg/mL); MBC: Minimal bactericidal concentration (μg/mL).

### Cytotoxic Activity

The cytotoxic activities of EO, WE, and EE were evaluated against human tumor cell lines (A549, NCI-H1299, PC-3, and K562), human normal cell line (MRC-5), and murine fibroblast cell line (L929) using the MTT method with cisplatin as positive control (**Table 4**). The EO showed higher inhibitory property on the test cell lines than WE and EE. Both WE and EE displayed very low cytotoxic activity against tumor and non-cancerous cell lines, indicating that WE and EE were less-toxic. The EO exhibited significant cytotoxicity against A549 (IC_50_ = 72.86 ± 6.39 μg/mL), NCI-H1299 (IC_50_ = 70.74 ± 9.56 μg/mL), PC-3 (IC_50_ = 63.16 ± 9.20 μg/mL), and K562 (IC_50_ = 27.16 ± 2.18 μg/mL) human tumor cell lines. Additionally, the EO displayed a considerable selectivity to human tumor cell K562, and its toxicity was more than 3.5-fold different from that of non-cancerous MRC-5 cell (IC_50_ = 95.96 ± 4.37μg/mL) and L929 cell (IC_50_ = 129.91 ± 5.27 μg/mL). According to previous studies, the coronarin E, as the most predominant constituent in *H. flavum* EO, had higher cytotoxicity to human tumor cell lines (NCI-H187, HL-60, THP-1, and A-549) (IC_50_: 31.21–53.26 μM) compared to non-cancerous Vero cell (IC_50_: 150.45 μM), with selective cytotoxicity against tumor cells (Reddy et al., 2009; Kumrit et al., 2010). The cytotoxic properties of other main components in EO, such as 1,8-cineole, *β*-pinene, *α*-pinene, *E*-nerolidol, linalool, and *α*-curcumene, have been demonstrated in previous reports (Hassan et al., 2010; Shin and Lee, 2013; Dall’Acqua et al., 2017; Salehi et al., 2019). Therefore, the presence of these main components could explain the significant cytotoxic activities of the *H. flavum* rhizome EO. These results indicate that the *H. flavum* EO can be used as a new source of natural antitumor active small molecules in the pharmaceutical industry.

**Table 4:** Cytotoxic activity of *H. flavum* rhizome EO, WE and EE using MTT assay.

Treatment	Cell line (IC_50_ μg/mL)^a^					
	MRC-5	L929	A549	NCI-H1299	PC-3	K562
EO	95.96 ± 4.37	129.91 ± 5.27	72.86 ± 6.39^c^	70.74 ± 9.56^c^	63.16 ± 9.20^c^	27.16 ± 2.18^c^
WE	nd	nd	nd	594.99 ± 25.68	nd	nd
EE	nd	nd	nd	679.66 ± 33.79	nd	nd
Cisplatin	0.95 ± 0.06	4.42 ± 0.14	4.73 ± 0.96	2.41 ± 0.66	0.37 ± 0.05	0.39 ± 0.08

a IC_50_: The sample concentration reduced cells growth by 50%. Cisplatin was used as positive control. Cell line: MRC-5 (human fetal lung fibroblasts cells), L929 (murine fibroblast cell line), PC-3 (human prostatic carcinoma cell line), A549 (human lung adenocarcinoma cell line), K562 (human leukemic cell line), NCI-H1299 (human non-small cell lung cancer cell line); nd, not determined at concentration <1000 μg/mL.

c Significantly diﬀerent from the normal cell line (MRC-5 and L929) (p < 0.05).

### EO Induces Apoptosis in K562 Cells

**Figure 4:**
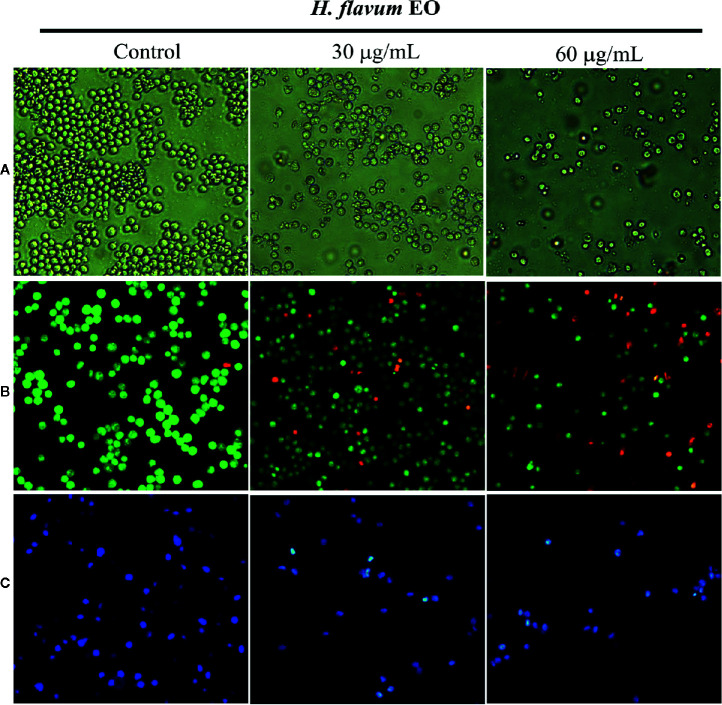
Effect of *H. flavum* EO on the morphology of K562 cells. **(A)** The cells were treated with EO (0, 30, 60 μg/mL) and the morphological changes of the cells were observed using an inverted microscope (100×). The cells were treated as described above and then stained with AO/EB **(B)** and Hoechst 33258 **(C)**. The morphological changes were observed using a fluorescence microscope (100×).

**Figure 5:**
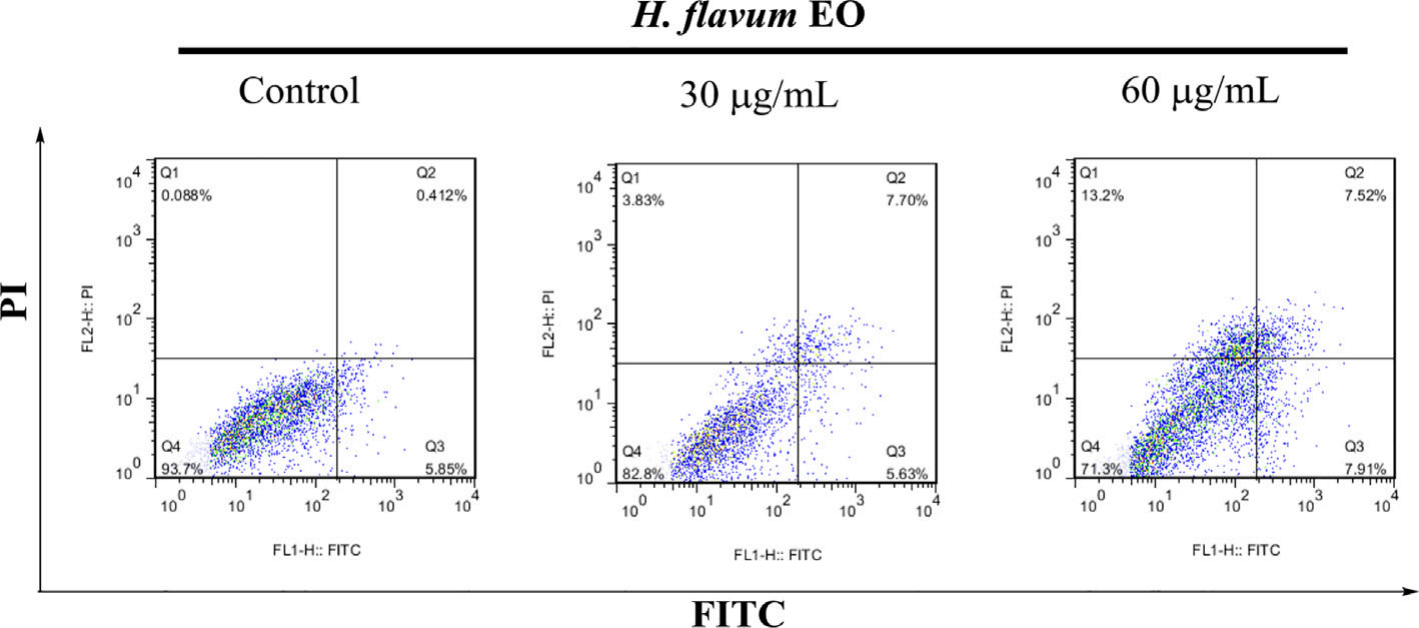
Apoptosis analysis of K562 cells treated with *H. flavum* EO using a flow-cytometer. Cells in upper left quadrant (Q1-UL: AV–/PI+): necrotic cells; upper right quadrant (Q2-UR: AV+/PI+): late apoptotic cells; lower right quadrant (Q3-LR: AV+/PI–): early apoptotic cells; lower left quadrant (Q4-LL: AV–/PI–): live cells.

Morphology assay indicated that EO triggered obvious morphological changes with typical characteristics of apoptosis, such as cell shrinkage and fragmentation in K562 cells (**Figure 4A**). AO/EB staining assay showed that the number of apoptotic cells with red fluorescence increased, while the number of viable cells with bright green fluorescence decreased when the cells were treated with increasing concentrations of EO (**Figure 4B**). Hoechst 33258 staining revealed that the nucleus of EO-treated cells presented more and brighter blue-fluorescence compared with the control group, which indicated typical apoptotic morphology (**Figure 4C**). Furthermore, to quantitatively determine the apoptotic effect of the EO, Annexin V-FITC/PI staining assay was performed using a flow-cytometer. As shown in **Figure 5**, the percentage of apoptotic cells (Q2+Q3) was enhanced to 13.72 ± 0.42% (at 30 μg/mL) and 15.48 ± 0.22% (at 60 μg/mL) in comparison to the control (6.14 ± 0.94%), which suggested that EO triggered apoptosis against K562 cells in a dose-dependent manner.

### Enzyme Inhibitory Activities

The inhibitory effect of *H. flavum* rhizome EO, WE, and EE on α-glucosidase, tyrosinase, and two cholinesterases (AChE and BChE) were investigated and the results are shown in **Table 5**.

**Table 5:** The enzyme inhibitory activity of *H. flavum* rhizome EO, WE, and EE^1^.

Samples	*α*-Glucosidase		Tyrosinase		Acetylcholinesterase		Butyrylcholinesterase	
	IC_50_ (mg/mL)	(mmoL ACEs/g sample)	IC_50_ (mg/mL)	(mg KAEs/g sample)	IC_50_ (mg/mL)	(mg GALAEs/g sample)	IC_50_ (mg/mL)	(mg GALAEs/g sample)
EO	4.15 ± 0.26^a^	0.094 ± 0.002^a^	6.12 ± 0.16^a^	32.51 ± 0.82^a^	4.84 ± 0.36^a^	0.051 ± 0.007^a^	23.66 ± 1.61^a^	0.20 ± 0.01^a^
WE	0.064 ± 0.004^b^	6.02 ± 0.79^b^	10.49 ± 1.48^b^	19.30 ± 2.72^b^	2.27 ± 0.13^b^	0.108 ± 0.011^b^	0.65 ± 0.04^b^	7.24 ± 0.26^b^
EE	0.19 ± 0.01^c^	2.04 ± 0.20^c^	9.62 ± 0.94^b^	20.85 ± 1.12^b^	5.93 ± 0.36^c^	0.042 ± 0.005^a^	16.64 ± 1.33^c^	0.23 ± 0.02^a^
Acarbose	0.25 ± 0.02^d^							
Kojic acid			0.20 ± 0.01^c^					
Galanthamine^*^					0.25 ± 0.04^d^		4.71 ± 0.15^d^	

^1^C_50_: The concentration of sample that affords a 50% inhibition in the assay. GALAEs, KAEs, and ACEs: galanthamine, kojic acid, and acarbose equivalents, respectively.

^a-d^Different letters in the same column represent significant difference (p<0.05).

*Galanthamine: IC_50_ (μg/mL).

**Figure 6:**
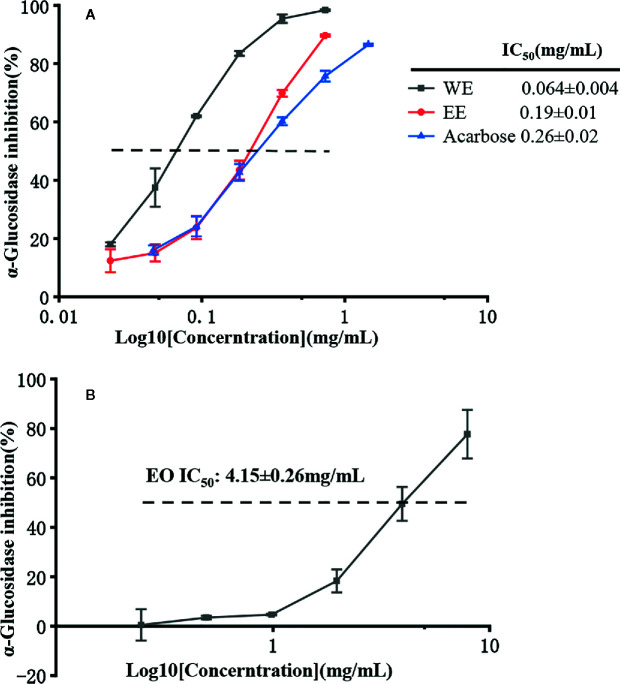
α-Glucosidase inhibitory activity of EO, WE, EE, and acarbose. **(A)** The inhibitions of WE, EE, and acarbose. **(B)** The inhibition of EO.

It is well known that α-glucosidase inhibitors reduce post-meal blood glucose and insulin levels by delaying the absorption of carbohydrates in the small intestine. Therefore, α-glucosidase inhibitors are used to treat type 2 diabetes (van de Laar et al., 2005). In this study, the inhibitory effect of α-glucosidase could be summarized with the following order: WE (IC_50_ = 0.064 ± 0.004 mg/mL) > EE (IC_50_ = 0.19 ± 0.01 mg/mL) > acarbose (IC_50_ = 0.25 ± 0.02 mg/mL) > EO (IC_50_ = 4.15 ± 0.26 mg/mL) (*p* < 0.05), which were presented in **Table 5** and **Figure 6**. *H. flavum* rhizome WE (6.02 ± 0.79 mmoL ACEs/g sample) and EE (2.04 ± 0.20 mmoL ACEs/g sample) showed higher α-glucosidase inhibitory capacity than EO (0.094 ± 0.002 ACEs/g sample), expressed as acarbose equivalents per gram of extract. Interesting findings in this study are that WE and EE had higher α-glucosidase inhibitory activity than acarbose. Hence, *H. flavum* rhizome WE and EE could be a promising α-glucosidase inhibitor, which could be developed as a therapeutic product for type 2 diabetes.

Tyrosinase is a key enzyme involved in the catalysis of mammalian melanogenesis and the enzymatic browning reaction in fruits and vegetables. Therefore, tyrosinase inhibitors are used to control enzymatic browning in foods and for treatment of hyperpigmentation in human skin (Chang, 2009). As shown in **Table 5**, the best inhibitory effect on tyrosinase was provided by the EO (IC_50_ = 6.12 ± 0.16 mg/mL, 32.51 ± 0.82 mg KAEs/g sample) followed by the EE (IC_50_ = 9.62 ± 0.94 mg/mL, 20.85 ± 1.12 mg KAEs/g sample) and WE (IC_50_ = 10.49 ± 1.48 mg/mL, 19.30 ± 2.72 mg KAEs/g sample). However, compared to positive control kojic acid (IC_50_ = 0.20 ± 0.01 mg/mL), all of EO, WE, and EE didn’t show a promising inhibition on tyrosinase.

Cholinesterase inhibitors have become the most effective treatment strategy for Alzheimer’s disease by inhibiting the breakdown of acetylcholine to enhance cholinergic neurotransmission (Grutzendler and Morris, 2001). As shown in **Table 5**, the WE was the most effective AChE inhibitor (IC_50_ = 2.27 ± 0.13 mg/mL, 0.108 ± 0.011 mg GALAEs/g sample) and BChE (IC_50_ = 0.65 ± 0.04 mg/mL, 7.24 ± 0.26 mg GALAEs/g sample). Compared with the positive control drug galanthamine, *H. flavum* rhizome EO, WE, and EE showed weak cholinesterase inhibitory activity.

According to previous studies, the IC_50_ values of α-glucosidase inhibitory effect of 1,8-cineole and *α*-pinene were 1.118 and 1.420 μg/mL, respectively (Basak and Candan, 2013). *α*-terpineol, *α*-pinene, and 1,8-cineole showed significant tyrosinase inhibitory effect (Chao et al., 2017). The significant inhibited AChE activity of 1,8-cineole, *β*-pinene, *α*-pinene, and linalool has been demonstrated in previous studies (Abdelgaleil et al., 2009; Kim et al., 2013; Seo et al., 2015). Additionally, the linalool-rich EO was reported to exhibit strong α-glucosidase, tyrosinase, and cholinesterases (AChE and BChE) inhibitory activity (Sarikurkcu et al., 2015). Therefore, the enzyme inhibitory activities of EO may be due to these main components.

Previous studies have indicated that phenolic compounds play an important role in enzyme inhibitory activity (Gonçalves and Romano, 2017). In this study, the α-glucosidase, and cholinesterases (AChE and BChE) inhibitory activities of WE were significantly higher than that of EE (p<0.05), except for not significant difference in tyrosinase inhibitory effect. However, WE revealed the significantly lower TPC and TFC compared with EE. This finding could be explained by the presence of non-phenolic and non-flavonoid enzyme inhibitors in the extract. Furthermore, UHPLC-Q-Orbitrap-MS analysis showed that many bioactive compounds may contribute to the enzyme inhibitory effect of the extract. For instance, the tyrosinase inhibitory activity of *p*-Coumaric acid (12) (IC_50_ = 0.66 μg/mL) was much higher than positive control arbutin (IC_50_ > 100 μg/mL), and *p*-Coumaric acid showed a moderate α-glucosidase inhibitory activity (Pei et al., 2016). According to Ma et al. (2020), carnosol (44) displayed a competitive inhibition (Ki =5.57 μg/mL) on α-glucosidase with IC_50_ value of 12.0 ± 0.8 μg/mL, and showed much stronger inhibitory effect than acarbose (IC_50_ = 412 ± 26 μg/mL). Ginkgolic acid (C13:0) (62) revealed higher α-glucosidase inhibitory activity (IC_50_ = 3.5 ± 0.13 μg/mL) than the positive control quercetin (IC_50_ = 3.8 ± 0.12 μg/mL) (Sukito and Tachibana, 2014).

## Conclusions

To our knowledge, the antioxidant, antibacterial, cytotoxic, and enzyme inhibitory activities of essential oil (EO), as well as the chemical composition and bioactivities of water extract (WE) and 70% ethanol extract (EE) from *H. flavum* rhizome were reported for the first time. The coronarin E was the most main constituent in EO and was first detected in *H. flavum*. The WE and EE exhibited high TPC and TFC values. Moreover, 86 compounds were putatively identified from WE and EE using UHPLC-Q-Orbitrap-MS, and all the compounds were reported in *H. flavum* for the first time. In addition to the low antioxidant effect of EO and the weak inhibitory activity of EO and extracts on tyrosinase and cholinesterase, EO demonstrated significant antibacterial activity against *P. vulgaris*, *P. aeruginosa*, *B. subtilis*, and *S. aureus*, and showed significant α-glucosidase inhibitory effect. Besides, EO exhibited a considerable selectivity to human tumor cell K562, and its toxicity was more than 3.5-fold different from that of non-cancerous MRC-5 and L929 cells. A series of apoptosis analysis suggested that EO triggered apoptosis against K562 cells in a dose-dependent manner. The very low inhibition against the tumor and non-cancerous cell lines suggested that WE and EE were less-toxic. Interesting findings in this study are that WE and EE displayed remarkable antioxidant and α-glucosidase inhibition activities, being superior to their respective positive control drugs BHT and acarbose. Hence, *H. flavum* could be considered as a source of bioactive compounds and has high exploitation potential in the cosmetics, food, and pharmaceutical industries.

## Data Availability Statement

The raw data supporting the conclusions of this article will be made available by the authors, without undue reservation, to any qualified researcher.

## Author Contributions

YZ and MT conceived and designed the experiments. MT and XW performed the experiments. TL, XZ, and FW analyzed the data. MT and GD drafted and revised the manuscript. All authors contributed to the article and approved the submitted version.

## Conflict of Interest

The authors declare that the research was conducted in the absence of any commercial or financial relationships that could be construed as a potential conflict of interest.
