# Supplementary material for: Phytochemical Analysis, Antioxidant, Antibacterial, Cytotoxic, and Enzyme Inhibitory Activities of Hedychium flavum Rhizome
Source: Front Pharmacol. 2020 Sep 17;11:572659. doi: 10.3389/fphar.2020.572659 (PMC7528636; doi:10.3389/fphar.2020.572659)
Supplement: Supplementary file 1 [file DataSheet_1.pdf]

SUPPLEMENTARY MATERIAL

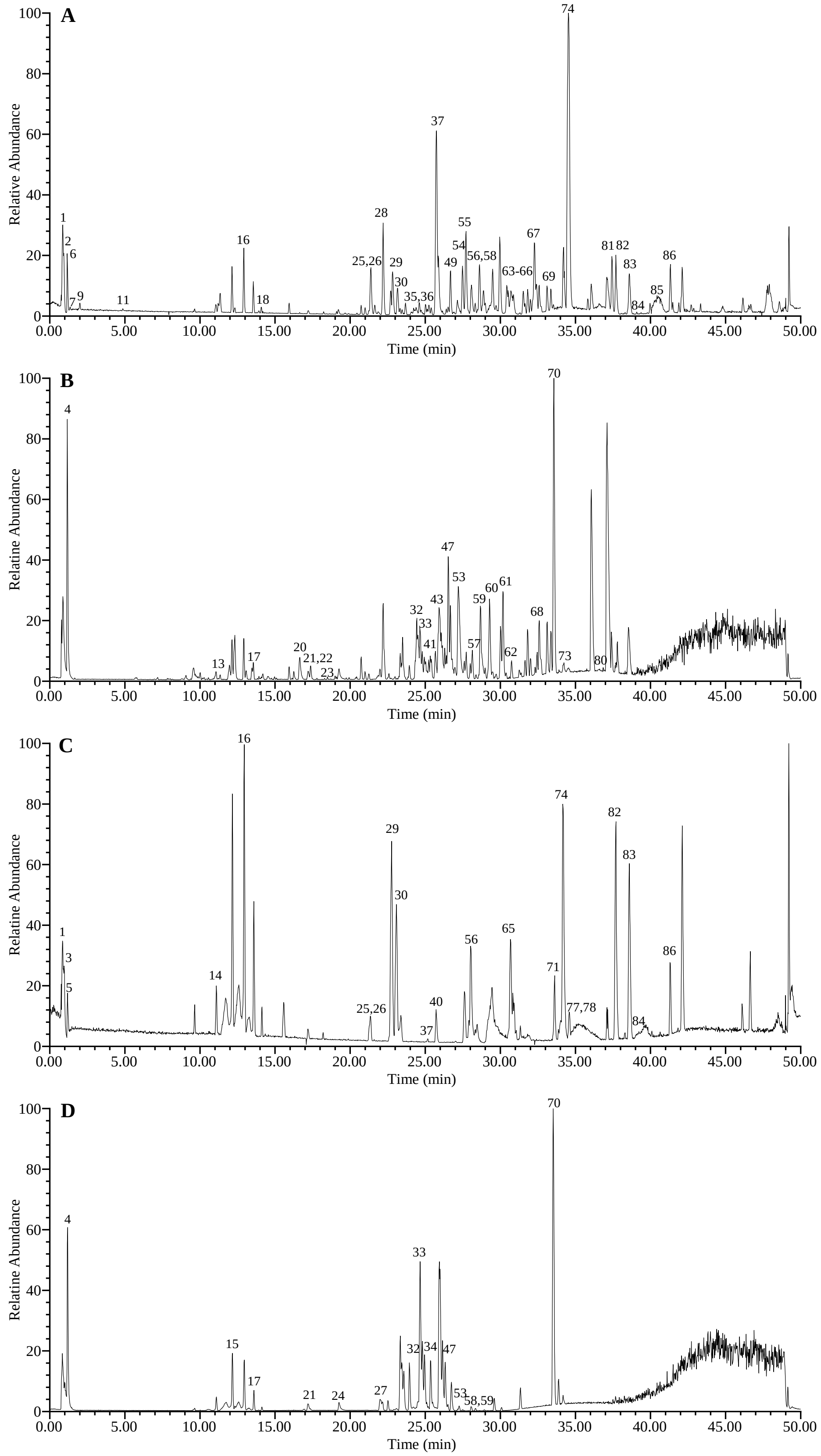

FIGURE S1. UHPLC-Q-Orbitrap-MS base peak chromatograms of *Hedychium flavum* rhizome water extract (WE) and 70% ethanol extract (EE). **(A)** WE in positive ion mode; **(B)** WE in negative ion mode; **(C)** EE in positive ion mode; **(D)** EE in negative ion mode.

Table S1 Phytochemical compounds detected and characterized in water extract (WE) and 70% ethanol extract (EE) of *Hedychium flavum* rhizome using UHPLC-Q-Orbitrap-MS in positive and negative ionization modes

Peak NO.	RT [min]	Identification[a]	Formula	$[M+H]^+$ (*m/z*)	$[M-H]^-$ (*m/z*)	Error ppm	MS^2 fragment ions	WE[b]	EE[b]
1	0.87	Choline[1]	$C_5H_{13}NO$	104.10749		4.8	104.10751, 60.08168	√	√
2	0.88	Pipecolic acid[1,3]	$C_6H_{11}NO_2$	130.08650		1.8	130.08640, 84.08152, 84.04508	√	-
3	0.91	DL-Arginine[1,3]	$C_6H_{14}N_4O_2$	175.11909		0.8	175.11888, 130.09750, 116.07082, 70.06594, 60.05647	-	√
4	1.17	Citric acid[1,2,3]	$C_6H_8O_7$		191.01906	-3.5	191.01892, 111.00755, 87.00747, 85.02820	√	√
5	1.23	Adenosine[1,2,3]	$C_{10}H_{13}N_5O_4$	268.10403		0.0	268.10370, 136.06187	-	√
6	1.24	L-Tyrosine[1,2,3]	$C_9H_{11}NO_3$	182.08131		0.8	165.05457, 136.07562, 123.04415, 119.04929, 91.05473	√	-
7	1.35	DL-Norleucine[1,3]	$C_6H_{13}NO_2$	132.10208		1.3	131.97426, 113.96394, 90.94824, 86.09707, 72.93790	√	-
8	1.39	L-(+)-Leucine[3]	$C_6H_{13}NO_2$	132.10210		1.4	131.97421, 113.96392, 90.94823, 86.09702, 72.93790	-	√
9	2.01	L-Phenylalanine[1,2,3]	$C_9H_{11}NO_2$	166.08643		1.0	166.08629, 120.08105, 103.05459	√	√
10	3.82	Indole-3-acrylic acid[1,3]	$C_{11}H_9NO_2$	188.07071		0.6	188.07045, 146.06001, 144.08069, 118.06533	√	-
11	4.86	Dropropizine[1,3]	$C_{13}H_{20}N_2O_2$	237.15988		0.5	237.15959, 160.09940, 120.08092	√	-
12	8.18	*p*-Coumaric acid[1,2]	$C_9H_8O_3$		163.03934	-4.5	163.01154, 119.04902	-	√
13	10.96	Cycloolivil[1,3]	$C_{20}H_{24}O_7$		375.14496	0.1	375.14471, 165.05475, 150.03119	√	-
14	11.25	Triethyl phosphate[1]	$C_6H_{15}O_4P$	183.07814		0.4	131.97423, 127.01554, 113.96393, 96.98460	-	√
15	12.36	(2'R,3R,4'R,4a'R,5S,8a'S)-5-(3-Furyl)-4'-hydroxy-4a',5'-bis(hydroxymethyl)-2'-methyl-3',4,4',4a',5,7',8',8a'-octahydro-2'H-spiro[furan-3,1'-naphthalen]-2-one[1,3]	$C_{20}H_{26}O_6$		361.16556	-0.3	361.16559, 135.04407	-	√
16	13.07	Ferulaldehyde[1,2,3]	$C_{10}H_{10}O_3$	179.07031		0.2	179.07048, 147.04396, 119.04932, 91.05474	√	√
17	13.46	1,7-Bis(4-hydroxyphenyl)-3,5-heptanediol[1]	$C_{19}H_{24}O_4$		315.16030	0.4	315.16025, 149.05981, 147.08052	√	√
18	13.92	Meconin[1,3]	$C_{10}H_{10}O_4$	195.06541		1.1	195.06514, 109.06512, 79.05489	√	-
19	16.24	Corchorifatty acid F[1,3]	$C_{18}H_{32}O_5$		327.21762	-0.2	327.21774, 229.14363, 211.13303	-	√
20	16.64	(-)-5'-Desmethylyatein[3]	$C_{21}H_{22}O_7$		385.12920	-0.2	385.12918, 370.10587, 179.03421, 164.01057	√	-
21	17.20	Monobutyl phthalate[1,3]	$C_{12}H_{14}O_4$		221.08153	-1.8	221.08136, 121.02833, 71.04895, 69.03329	√	√
22	17.36	(15Z)-9,12,13-Trihydroxy-15-octadecenoic acid[1,3]	$C_{18}H_{34}O_5$		329.23340	0.2	329.23325, 229.14398, 211.13338	√	√
23	18.70	Ingenol [1,2,3]	$C_{20}H_{28}O_5$		347.18631	-0.3	347.18665, 329.17697, 135.00746	√	-
24	19.51	16,17-Dihydroxykauran-18-oic acid[1,3]	$C_{20}H_{32}O_4$		335.22275	-0.1	335.22287, 317.21350, 259.20761	-	√
25	21.38	2-Methoxyestradiol[1,3]	$C_{19}H_{26}O_3$	303.19550		0.1	303.19534, 285.18469, 179.07019	√	√
26	21.38	N-(4-cyano-1-phenyl-1H-pyrazol-5-yl)-2-(4-methylpiperazino)acetamide[1,3]	$C_{17}H_{20}N_6O$	325.17740		0.8	325.17709	√	√
27	22.14	Dodecyl sulfate[1]	$C_{12}H_{26}O_4S$		265.14777	-0.5	265.14767, 96.95879	-	√
28	22.23	Kahweol[1,2,3]	$C_{20}H_{26}O_3$	315.19534		-0.4	315.19531, 191.07019, 145.06483, 139.03893, 117.07012	√	-
29	22.71	Bis(4-ethylbenzylidene)sorbitol[1,3]	$C_{24}H_{30}O_6$	415.21188		0.9	119.08584, 91.05469	√	√
30	23.16	2-Amino-1,3,4-octadecanetriol[1]	$C_{18}H_{39}NO_3$	318.30057		0.9	318.30002, 256.26318, 102.09171, 88.07625, 70.06586	√	√
31	24.07	7*α*-Hydroxytestosterone[1,3]	$C_{19}H_{28}O_3$	305.21121		0.3	305.21091, 287.20035, 95.08601, 81.07051	√	-
32	24.50	Prostaglandin F2*α* 1-11-lactone[1,3]	$C_{20}H_{32}O_4$		335.22280	0.1	335.22284, 273.22247, 69.03328	√	√
33	24.66	4-Dodecylbenzenesulfonic acid[1,3]	$C_{18}H_{30}O_3S$		325.18430	0.0	325.18433, 183.01138	√	√
34	25.09	Myristyl sulfate[1]	$C_{14}H_{30}O_4S$		293.17917	-0.1	293.17908, 96.95879	-	√
35	25.13	(7E,13E)-11,12-Dihydroxy-3-isobutyl-4,5,8-trimethyl-3,3a,4,6a,9,10,11,12-octahydro-1H-cycloundeca[d]isoindole-1,15(2H)-dione[1,3]	$C_{24}H_{35}NO_4$	402.26425		0.9	402.26361, 210.07607, 192.06551, 177.16370, 164.07051, 98.06048, 95.08599	√	-
36	25.39	17*β*-Hydroxy-androstano[3,2-c]isoxazole[1]	$C_{20}H_{29}NO_2$	316.22723		0.4	316.22690, 177.16366, 124.03942, 96.04486, 95.08600	√	-

37	25.74	(+)-Nootkatone[1,2,3]	$C_{15}H_{22}O$	219.17430		-0.2	219.17419, 149.09601, 95.08598, 81.07050	√	√
38	25.74	Perillene[1,2,3]	$C_{10}H_{14}O$	151.11177		0.2	151.11172, 123.11696, 109.06511, 81.07053	√	-
39	25.74	Valerophenone[1,3]	$C_{11}H_{14}O$	163.11168		-0.4	163.11168, 145.10114, 121.06496, 107.08588	√	-
40	25.76	Triphenyl phosphate[1]	$C_{18}H_{15}O_4P$	327.07785		-0.7	327.07782, 233.03609, 152.06201, 95.04961	√	√
41	25.79	1-(3-Cyanophenyl)-3-{[(2R,4S,5R)-5-(3-cyclopentyl-1-methyl-1H-pyrazol-5-yl)-1-azabicyclo[2.2.2]oct-2-yl] methyl}urea[1]	$C_{25}H_{32}N_6O$	433.26932		-4.0	431.25562, 316.22809, 114.01852, 110.02351	√	-
42	25.91	3,5-di-tert-Butyl-4-hydroxybenzaldehyde[1,3]	$C_{15}H_{22}O_2$	235.16925		0.0	235.16908, 179.10661, 57.07072	-	√
43	26.07	5-[2-(3-Furyl)ethyl]-8a-(hydroxymethyl)-5,6-dimethyl-3,4,4a,5,6,7,8,8a-octahydro-1-naphthalenecarboxylic acid[1,3]	$C_{20}H_{28}O_4$		331.19162	0.4	331.19150, 287.20172	√	√
44	26.45	Carnosol[2,3]	$C_{20}H_{26}O_4$		329.17587	0.1	329.17572, 257.19086, 65.00197	√	-
45	26.51	Testosterone[1,3]	$C_{19}H_{28}O_2$	289.21628		0.2	289.21594, 243.21046,107.08588, 95.08600, 81.07048	√	-
46	26.51	Cafestol[1,2,3]	$C_{20}H_{28}O_3$	317.21121		0.3	317.21078, 299.20016, 271.20523, 123.11700, 95.08605, 81.07053	√	-
47	26.54	13,14-Dihydro-15-keto Prostaglandin A2[1,3]	$C_{20}H_{30}O_4$		333.2072	0.2	333.20709, 289.21732	√	√
48	26.54	5*α*-Dihydrotestosterone[1,3]	$C_{19}H_{30}O_2$		289.21725	-0.2	289.21732, 59.98389	√	-
49	26.58	*α*-Cyperone[1,2,3]	$C_{15}H_{22}O$	219.17438		0.2	219.17426, 204.15080, 189.12726, 111.08070	√	-
50	26.63	(+/-)12(13)-DiHOME[1,3]	$C_{18}H_{34}O_4$		295.22772 $[M-H_2O-H]^-$	-0.5	295.22775, 277.21732, 195.13860, 171.10170	√	-
51	27.07	(1S,4aR,5S)-5-[(3E)-5-Methoxy-3-methyl-5-oxo-3-penten-1-yl]-1,4a-dimethyl-6-methylenedecahydro-1-naphthalenecarboxylic acid[1,3]	$C_{21}H_{32}O_4$		347.22281	0.1	347.22269, 303.23288, 59.01266	√	-
52	27.15	(E,E)-*α*-Farnesene[3]	C15 H24	205.1952		0.6	205.19508, 149.13251, 121.10134, 109.10153,95.08604, 93.07042	√	-
53	27.29	5-(4-Carboxy-3-methylbutyl)-1,4a-dimethyl-6-methylenedecahydro-1-naphthalenecarboxylic acid[1,3]	$C_{20}H_{32}O_4$		335.22281	0.1	335.22281, 317.21222, 291.23303	√	√
54	27.48	Isosteviol[1,2,3]	$C_{20}H_{30}O_3$	319.22681		0.1	319.22650, 191.17920, 109.10156, 95.08598, 81.07050	√	-
55	27.73	2-Amino-1,3-octadecanediol[1,3]	$C_{18}H_{39}NO_2$	302.30539		0.1	302.30515, 302.21948, 109.10153, 95.10153, 88.07626, 81.07049	√	-
56	28.07	Diisobutylphthalate[1,3]	$C_{16}H_{22}O_4$	279.15918		0.3	149.02333, 121.02879, 111.08749, 95.08597,81.07059	√	√
57	28.17	Oleoyl-L-*α*-lysophosphatidic acid[1]	$C_{21}H_{41}O_7P$		435.25156	-0.3	435.25241, 152.99487, 78.95774	√	-
58	28.34	Prostaglandin F2*α* 1-11-lactone[1,3]	$C_{20}H_{32}O_4$		317.21210 $[M-H_2O-H]^-$	-0.4	317.21216,273.22217,80.30635	-	√
59	28.68	15-Deoxy-*δ*12,14 -Prostaglandin J2[1,3]	$C_{20}H_{28}O_3$		315.19670	0.4	315.19666, 289.21701, 271.20682, 269.19113, 197.09660	√	√
60	28.96	5-[(2Z,8Z)-2,8-Pentadecadien-1-yl]-1,3-benzenediol[1,3]	$C_{21}H_{32}O_2$		315.23297	0.1	315.23288, 271.20728, 269.19077, 197.09683, 67.05391	√	-
61	30.18	(5*ξ*,9*ξ*)-17-Hydroxykaur-15-en-19-oic acid[1,3]	$C_{20}H_{30}O_3$		317.21230	0.3	317.21222, 273.22238, 69.03329	√	-
62	30.27	Ginkgolic acid (C13:0)[1,2]	$C_{20}H_{32}O_3$		319.22787	0.0	319.22778, 275.23779	√	-
63	30.37	(-)-Caryophyllene oxide[1,3]	$C_{15}H_{24}O$	221.18997		-0.1	221.18990, 203.17940, 163.14801, 147.11679, 109.10148, 107.08588, 95.08598, 81.07053, 59.05000	√	-
64	30.55	Trihexyphenidyl[1]	$C_{20}H_{31}NO$	302.24789		0.2	302.24799, 138,09120, 109.10149, 96.04486, 95.08602, 81.07053	√	-
65	30.71	Citroflex A-4[1]	$C_{20}H_{34}O_8$	403.23276		0.3	259.15332, 185.08076, 157.01312, 139.00240, 129.01831, 68.99781	√	√
66	32.00	Laurophenone[3]	$C_{18}H_{28}O$	261.22137		0.3	261,22119, 243.21053, 95.08601, 81.07050	√	-
67	32.27	Abietic acid[1,2,3]	$C_{20}H_{30}O_2$	303.23206		0.7	303.23169, 111.04437, 95.08601, 81.07053	√	-
68	32.45	Arachidonic acid[1,3]	$C_{20}H_{32}O_2$		303.23297	0.1	303.23288, 257.22803, 57.34408	√	-
69	33.53	N-(4-Pyridinyl)-N'-(1,2,3,4-tetrahydro-9-acridinyl)-1,7-heptanediamine[1,3]	$C_{25}H_{32}N_4$	389.26877		-3.1	389.26794, 371.25729, 343.26245, 217.19502, 109.10151, 95.08603	√	-
70	33.57	2,2'-Methylenebis(4-methyl-6-tert-butylphenol)[1]	$C_{23}H_{32}O_2$		339.23280	-0.4	339.23285, 163.11197	√	√
71	33.61	1-Palmitoylglycerol[1,3]	$C_{19}H_{38}O_4$	331.28409		-0.6	313.27365 ,95.08598, 71.08627, 57.07076	-	√

72	34.20	Muscone[1,3]	$C_{16}H_{30}O$	239.23677		-0.7	95.08601, 81.07049, 71.08625, 57.07074	√	√
73	34.23	10(E),12(Z)-Conjugated linoleic acid[1,3]	$C_{18}H_{32}O_2$		279.23291	-0.1	279.23288, 88.87541, 68.23261, 65.67889	√	-
74	34.53	Isotretinoin[1,3]	$C_{20}H_{28}O_2$	301.21643		0.7	301.21606, 123.11695, 109.10153, 95.08602, 81.07052	√	√
75	34.54	Docosahexaenoic acid[1,3]	$C_{22}H_{32}O_2$	329.24725		-0.8	329.24728, 283.20535, 255.21053, 163.14809, 95.08601	√	-
76	34.55	2,7-Dihydroxycadalene[3]	$C_{15}H_{18}O_2$	231.13782		-0.6	154.09641, 89.06027, 71.08627, 57.07074	√	-
77	34.59	Oleamide[1,3]	$C_{18}H_{35}NO$	282.27924		0.4	282.27896,247.24191,97.10164,83.08616,69.07064	-	√
78	34.59	Hexadecanamide[1]	$C_{16}H_{33}NO$	256.26355		0.2	256.26324,102.09165,88.07623,71.08621,57.07069	-	√
79	35.18	Ciprostene[1,3]	$C_{22}H_{36}O_4$	347.25821[M-H_2O+H]$^+$		0.4	347.25821, 301.21704, 273.22113, 257.22629, 255.21086	√	-
80	36.67	Elaidic acid[1,3]	$C_{18}H_{34}O_2$		281.24863	0.1	281.24860	√	-
81	37.43	(9cis)-Retinal[1,3]	$C_{20}H_{28}O$	285.22128		0.0	285.22101, 267.21066, 95.08603, 93.07033, 81.07050	√	-
82	37.69	1-Stearoylglycerol[1,3]	$C_{21}H_{42}O_4$	359.31555		-0.1	341.30475, 95.08595, 85.10179, 71.08624, 57.07073	√	√
83	38.59	Stearamide[1]	$C_{18}H_{37}NO$	284.29480		0.0	284.29462, 116.10714, 102.09164, 57.07071	√	√
84	39.37	Tridemorph[1]	$C_{19}H_{39}NO$	298.31046		0.1	298.31027, 116.10732, 102.09176, 58.02952,57.07069	√	√
85	40.53	(22E)-Stigmasta-5,22-dien-3-ol[3]	$C_{29}H_{48}O$	413.37772		-0.2	413.37747, 123.08053, 109.06512, 97.06527, 81.07049	√	-
86	41.32	Erucamide[1]	$C_{22}H_{43}NO$	338.34171		-0.1	338.34125, 321.31555, 303.30457, 97.10162, 83.08615, 69.07061	√	√

Notes: Data collection time (47–50 min) was to equilibrate the column, and the corresponding compounds were not listed.

[a] Identification: Based on comparison with mzCloud (1), mzVault (2), and ChemSpider (3) databases.

[b] "√" mean detected from extracts, "-" means undetected from extracts.
